# “Call for Action” for the challenges of kidney diseases in aging/aged societies

**DOI:** 10.1007/s10157-024-02591-7

**Published:** 2024-11-15

**Authors:** Masaomi Nangaku, Yusuke Suzuki, Motoko Yanagita

**Affiliations:** 1 Division of Nephrology and Endocrinology, The University of Tokyo Graduate School of Medicine, Tokyo, Japan; 2 Department of Nephrology, Juntendo University Graduate School of Medicine, Tokyo, Japan; 3 Department of Nephrology, Kyoto University Graduate School of Medicine, Kyoto, Japan

Japanese Society of Nephrology (JSN) and European Rena Association (ERA) have maintained a collaborative relationship since the establishment of their agreement in 2015. Japan is recognized as one of the world's most aged societies, with many European countries also experiencing demographic aging. As populations age, kidney health and disease are expected to become increasingly important; consequently, JSN and ERA, in collaboration with the Japanese Society for Dialysis Therapy (JSDT), organized a symposium titled “Kidney Health in Aging and Aged Societies” on September 14–15, 2024, in Kyoto, Japan. The symposium comprised six sessions addressing world demographic changes and kidney disease in aging/aged societies, healthy aging and the kidney, mechanisms of aging, unique comorbidities and their management in aged CKD patients, kidney replacement therapy in the elderly, and the future of aging/aged societies: high-tech assistive devices and policy development. Experts from both societies and external societies delivered comprehensive lectures, followed by robust discussions involving the attendees. The symposium attracted more than 700 participants, including more than 160 international attendees. From a total of 268 abstracts submitted, 19 abstracts were selected for JSN/ERA Symposium Travel Grant Award 2024, six abstracts were designated as JSN/ERA Symposium Award 2024—Outstanding Abstract Award, and three presentations were chosen for JSN/ERA Symposium Award 2024–Best Presentation Award among presentations of the Outstanding Abstract Award.

**Fig. 1:**
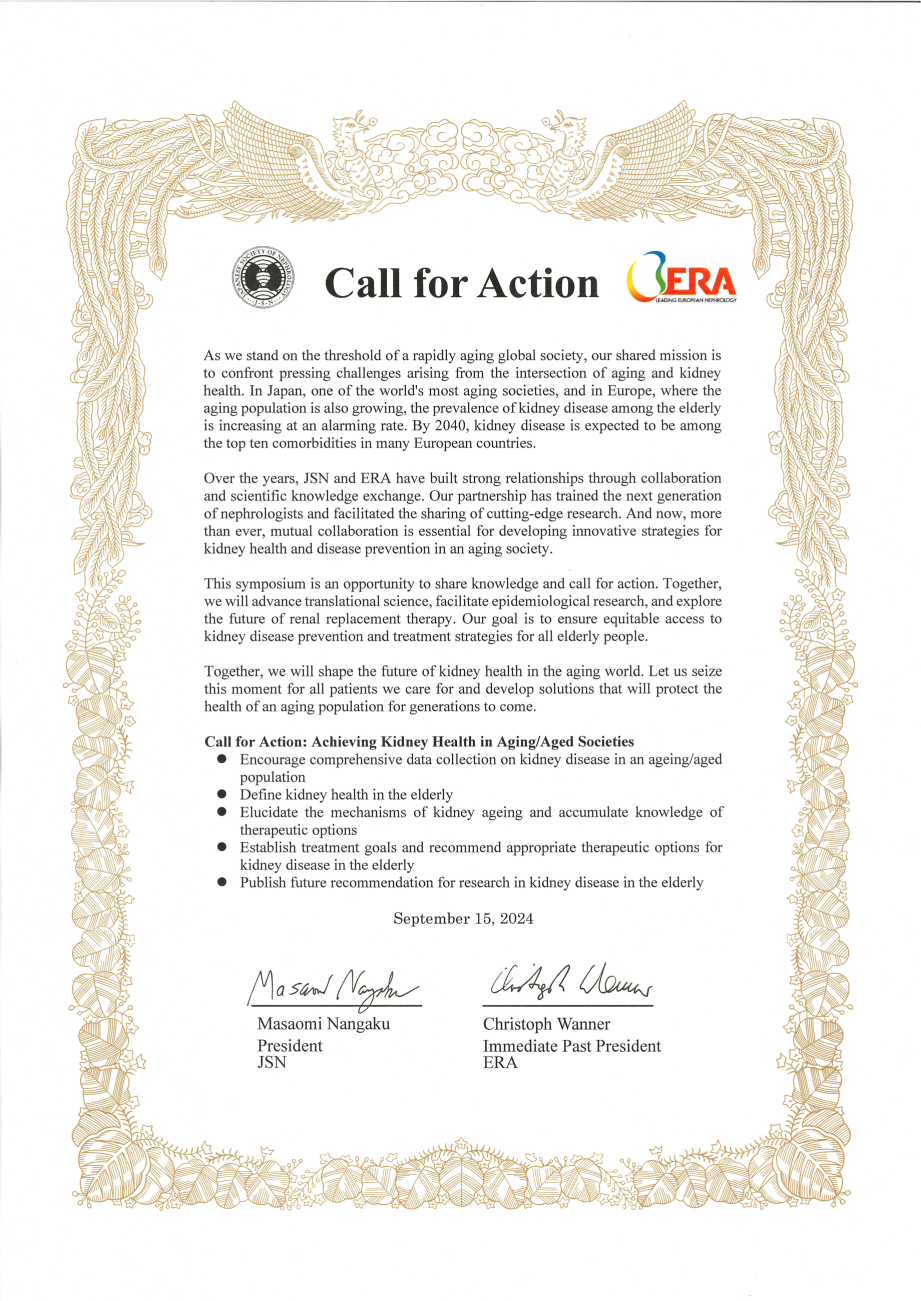
JSN&ERA signed_Call for Action

During the closing ceremony, Congress Chairs, Professor Wanner and President Nangaku presented the Call for Action, as shown in Fig. 1, and affirmed that both societies would implement measures to address the challenges of kidney diseases in aging/aged societies based on this Call for Action. The symposium's engaging discussions and friendships fostered during the symposium are anticipated to serve as a foundation for future collaboration between the two societies.

In organizing this symposium, the local organizing committee (LOC) members led by Yusuke Suzuki, LOC Chair, and Hideki Yokoi, Secretary General, made great contributions to the implementation of the symposium. In addition, we would like to express our deepest gratitude to Immediate Past President Kashihara for his valuable suggestions on the overall preparation and management of the symposium.

Congress Chair and President of Japanese Society of Nephrology: Masaomi Nangaku

Local Organizing Committee Chair: Yusuke Suzuki

Program Committee Chair: Motoko Yanagita

